# Effects of tonotopic matching and spatial cues on segregation of competing speech in simulations of bilateral cochlear implants

**DOI:** 10.1371/journal.pone.0270759

**Published:** 2022-07-05

**Authors:** Mathew Thomas, Shelby Willis, John J. Galvin, Qian-Jie Fu

**Affiliations:** 1 Department of Head and Neck Surgery, David Geffen School of Medicine, UCLA, Los Angeles, CA, United States of America; 2 House Institute Foundation, Los Angeles, California, United States of America; University of Florida, UNITED STATES

## Abstract

In the clinical fitting of cochlear implants (CIs), the lowest input acoustic frequency is typically much lower than the characteristic frequency associated with the most apical electrode position, due to the limited electrode insertion depth. For bilateral CI users, electrode positions may differ across ears. However, the same acoustic-to-electrode frequency allocation table (FAT) is typically assigned to both ears. As such, bilateral CI users may experience both intra-aural frequency mismatch within each ear and inter-aural mismatch across ears. This inter-aural mismatch may limit the ability of bilateral CI users to take advantage of spatial cues when attempting to segregate competing speech. Adjusting the FAT to tonotopically match the electrode position in each ear (i.e., increasing the low acoustic input frequency) is theorized to reduce this inter-aural mismatch. Unfortunately, this approach may also introduce the loss of acoustic information below the modified input acoustic frequency. The present study explored the trade-off between reduced inter-aural frequency mismatch and low-frequency information loss for segregation of competing speech. Normal-hearing participants were tested while listening to acoustic simulations of bilateral CIs. Speech reception thresholds (SRTs) were measured for target sentences produced by a male talker in the presence of two different male talkers. Masker speech was either co-located with or spatially separated from the target speech. The bilateral CI simulations were produced by 16-channel sinewave vocoders; the simulated insertion depth was fixed in one ear and varied in the other ear, resulting in an inter-aural mismatch of 0, 2, or 6 mm in terms of cochlear place. Two FAT conditions were compared: 1) clinical (200–8000 Hz in both ears), or 2) matched to the simulated insertion depth in each ear. Results showed that SRTs were significantly lower with the matched than with the clinical FAT, regardless of the insertion depth or spatial configuration of the masker speech. The largest improvement in SRTs with the matched FAT was observed when the inter-aural mismatch was largest (6 mm). These results suggest that minimizing inter-aural mismatch with tonotopically matched FATs may benefit bilateral CI users’ ability to segregate competing speech despite substantial low-frequency information loss in ears with shallow insertion depths.

## Introduction

In the clinical fitting of cochlear implants (CIs), the lowest acoustic input frequency is typically much lower than the characteristic frequency associated with the most apical electrode position [1], resulting in some degree of frequency mismatch (“tonotopic mismatch”). Previous studies have shown that tonotopic mismatch negatively affects speech recognition in CI listeners [2, 3] and in normal-hearing (NH) subjects listening to acoustic CI simulations [4]. One approach to reducing tonotopic mismatch would be to adjust the frequency allocation table (FAT) according to the actual intra-cochlear electrode position, which can be estimated from surgical imaging data using the Greenwood [5] frequency-place formula. The largest tonotopic mismatch typically occurs in the most apical region of the cochlea, where important low-frequency speech cues are delivered. Adjusting the FAT according to the actual electrode position may reduce tonotopic mismatch in the apical region. However, increasing the low input acoustic frequency to match the characteristic frequency associated with the most apical electrode position inevitably results in the loss of the low-frequency information below the new modified frequency, which may also negatively affect speech understanding on its own. For example, if the characteristic frequency of the most apical electrode is 500 Hz, adjusting the input acoustic frequency to 500 Hz would eliminate all speech information below 500 Hz. This trade-off between tonotopic mismatch and information loss has been well documented in unilateral CI users and NH subjects listening to acoustic CI simulations [2, 6].

For bilateral CI users, the electrode position may differ across ears. However, during the clinical fitting process, the same FAT is typically assigned to these electrodes in each ear. As such, in addition to the *intra-aural* tonotopic mismatch in each ear, bilateral CI users may also experience *inter-aural* mismatch, where the same input frequency stimulates a different cochlear place across ears. Inter-aural mismatch has been shown to limit binaural performance and benefits in bilateral CI users [7, 8] and in NH subjects listening to acoustic simulations of bilateral CIs [9, 10]. Furthermore, inter-aural mismatch has been shown to limit the perception of auditory inter-aural time and level differences [7, 11], which in turn can impact sound localization [12] and speech understanding in the presence of spatially separated maskers [9, 10].

Symmetrically placed speech maskers have been used to estimate spatial masking release, relative to co-located target and masker speech. These spatial configurations may be implemented in sound fields via loudspeaker presentations or in virtual space via a modified headphone presentation. In the case of a headphone presentation, the difference between diotic and dichotic masker presentation have been used to estimate spatial masking release [10]. However, dichotic masker presentation via headphones may overestimate spatial masking release in NH listeners compared to presentations in a sound field with symmetrically placed maskers [13–17]. Head-related transfer functions (HRTFs) may help to compensate for differences in spatial masking release between headphone and sound field presentations [18].

Since it is difficult to estimate the functional spectral resolution and degree of intra- and inter-aural mismatch in bilateral CI users, acoustic simulations of bilateral CIs in NH listeners have been used to parametrically control the spectral resolution and the electrode position in each ear. Such bilateral CI simulations necessitate the headphone presentation style to deliver the signal processing independently to each ear. In a previous study, Xu et al. [10] used diotic and dichotic masker presentation to measure segregation of competing speech in NH participants listening to bilateral CI simulations. They found that inter-aural mismatch had a relatively small effect on segregation for diotic presentation (i.e., co-located target and maskers), but had a much more negative effect on segregation for dichotic presentation (i.e., spatially separated target and maskers). Spatial masking release was found to worsen as the spectral resolution was reduced. In a related study, Hu et al. [18] measured the segregation of competing speech in NH participants listening to acoustic bilateral CI simulation (via headphone presentation) and in bilateral CI users (via direct audio input to each CI); HRTFs were used, rather than the diotic/dichotic masker presentation in Xu et al. [10]. Maskers were co-located with the target or symmetrically placed at ±60° azimuth; for the symmetrically placed maskers, signal processing was also applied to create an “infinite” inter-aural level difference (ILD), similar to the dichotic presentation in Xu et al. [10]. They found that spatial release from masking was poorer in bilateral CI users and in the bilateral CI simulations, compared to NH participants listening to unprocessed speech, similar to the effects of reducing the spectral resolution in Xu et al. [10]. In fact, some studies have even reported negative spatial release from masking (i.e., poorer SRTs with dichotic than with diotic presentation) in bilateral CI users [19, 20]. Presumably, poor spectral resolution limited the benefit of the spatial cues available during the segregation of competing speech.

In Xu et al. [10], the degree of inter-aural mismatch was parametrically manipulated (0-, 1-, or 2-mm mismatch), and spatial masking release was shown to monotonically decrease from 7.6 to 1.7 dB as the inter-aural mismatch was increased. This suggests that spatial release from masking is very sensitive to even a small degree of inter-aural mismatch. However, some bilateral CI users may have much larger differences in electrode insertion depths, resulting in even greater inter-aural mismatch [8]. It remains unclear how spatial masking release may be affected by large inter-aural mismatches. Of note, spatial masking release was likely overestimated with the dichotic masker presentation in Xu et al. [10]; Hu et al. [18] found that spatial release from masking was 6 dB larger with the infinite ILD, compared to symmetrically placed maskers at ±60°.

As noted above, adjusting the FAT to match the characteristic frequency associated with the electrode position may reduce intra- and inter-aural mismatch, but also introduces low-frequency information loss in one or both ears. The amount of low-frequency information loss largely depends on the insertion depth, with shallower insertion depths resulting in larger low-frequency information loss. For unilateral CI users with a shallow insertion depth, adjusting the FAT may not be an ideal approach, as the deficits associated with low-frequency information loss tend to overshadow the benefits of reducing the intra-aural mismatch. However, for bilateral CI users with different insertion depths across ears, the greater low-frequency information loss in the CI ear with the shallower insertion may be less detrimental to bilateral speech understanding, as there will be less information loss in the ear with the deeper insertion. The tradeoff between tonotopic matching and information loss has not been widely studied in bilateral CI users or in NH participants listening to acoustic simulations of bilateral CIs. Moreover, it is unclear how tonotopic matching may help bilateral CI users take advantage of spatial masking cues. While bilateral CI users receive some of the benefits that come with binaural hearing, they often have difficulty with binaural fusion, i.e., fusing sounds from the two ears into a single percept [12]. Reducing inter-aural mismatch may help fuse the target speech into a more coherent binaural image. However, adjusting the FAT in two ears with different insertion depths would likely result in different spectral patterns across ears, especially in the apical frequency regions. Staisloff et al. [21] found that perceptually aligning apical frequency regions generally lead to better binaural fusion outcomes of speech in an acoustic bilateral CI simulation. As such, there may be another tradeoff between improved binaural fusion via reduced inter-aural mismatch and misalignment of apical frequency regions across ears, possibly limiting the benefit of inter-aural matching.

The goal of the present study was to evaluate the effects of tonotopic matching on the segregation of competing speech, where target and masker speech was either co-located or spatially separated. NH listeners were tested while listening to acoustic simulations of bilateral CIs via headphones. Unlike the diotic-dichotic masker presentation in Xu et al. [10], HRTFs were used to more accurately simulate head shadowing effects associated with a sound field presentation. In the bilateral CI simulations, the insertion depth in each ear was parametrically manipulated. In one condition, the FAT in each ear was tonotopically matched to the insertion depth, resulting in increased low-frequency information loss as the insertion depth was reduced. In another condition, a fixed wide-band FAT was used in both ears without regard to the differences in insertion depths across ears, similar to the clinical fitting process of bilateral CIs. This approach preserved low-frequency information in each ear, but also resulted in substantial inter-aural mismatch when the insertion depths differed across ears. We hypothesized that reducing inter-aural mismatch would benefit the segregation of competing speech, despite the loss of low-frequency speech information in the ear with a shallower insertion depth.

## Materials and methods

### Ethics statement

This study was approved by the Institutional Review Board of the University of California, Los Angeles (UCLA IRB#19–000722) and this research was conducted in accordance with the principles of the Declaration of Helsinki and its later amendments. Prior to participation, written informed consent was obtained from all participants, in accordance with a protocol approved by the Institutional Review Board at the University of California, Los Angeles. All participants were given the opportunity to ask questions pertaining to the safety and modality by which the study would be conducted.

### Participants

Twenty NH adults (5 males and 15 females; mean age = 35.5 yrs) participated in the study. All participants had pure tone thresholds < 25 dB hearing loss (HL) at all audiometric frequencies between 250 and 8000 Hz in both ears. The participants were a random cohort with no known affiliations between any subjects and all participants were compensated upon completion.

### Test materials

Speech reception thresholds (SRTs), defined as the target-masker ratio (TMR) that produces 50% correct recognition of keywords in competing speech, were measured using a closed-set, matrix-styled test paradigm [22]. Target and masker speech consisted of five-word sentences that were constructed for each test trial by randomly selecting one of ten words from each of five categories (Name, Verb, Number, Color, and Clothing). The target sentence was produced by a male talker [mean fundamental frequency (F0) across all 50 words = 106 Hz] and two different masker sentences were produced by two different male talkers (mean F0 = 128 Hz and 97 Hz). The target sentence was always cued by the first word “John” and all five words were mutually exclusive among the target and masker sentences. A more detailed description regarding the test materials can be found in Xu et al. [10] and Willis et al. [19].

### Bilateral CI simulation signal processing

All target and masker stimuli were generated in real-time and delivered to circumaural headphones (Sennheiser HDA 200) via an audio interface (Edirol UA-25EX) connected to a mixer (Mackie 402). Non-individualized HRTFs were also used to create a virtual auditory space for headphone presentation of the stimuli [23]. The target sentence always originated directly in front of the listener (0° azimuth), and the two masker sentences were either co-located with the target (0°) or spatially separated from the target (±90°).

In each test trial, the target and masker sentences were first generated according to the specified TMR. The TMR was calculated according to long-term root-mean-squared (RMS) power between the target sentence and masker sentences. The target and masker sentences were first processed by the HRTF and then mixed independently into the left and right channel. The mixed target and masker sentences in each channel were then processed by a 16-channel sine-wave vocoder [2]. First, the signal was processed through a high-pass pre-emphasis filter with a cutoff of 1200 Hz and a slope of -6 dB/octave. The input frequency range was then divided into 16 frequency analysis bands according to the experimental FAT using 4th-order Butterworth filters that were distributed according to Greenwood’s [5] frequency-place formula. The temporal envelope from each analysis band was extracted using half-wave rectification and low-pass filtering (cutoff frequency = 160 Hz). Next, the extracted envelopes were used to modulate the amplitude of sinewave carriers. The distribution of the carrier sinewaves assumed a 20-mm electrode array with 16 electrodes linearly spaced in terms of cochlear place. Three different insertion depths were simulated: 27, 25, and 21 mm, relative to the base. Note that the cochlear extent (frequency bandwidth) of the simulated electrode array was held constant, and only the simulated insertion depth was manipulated. The simulated insertion depths represented a mild, moderate, or very large tonotopic mismatch between the acoustic input frequency and the expected spiral ganglion characteristic frequency associated with the CI electrode position. The carrier frequency range was always fixed in the left ear to simulate a 27-mm insertion depth, and was varied in the right ear to simulate a 27-, 25-, or 21-mm insertion depth. As such, there was an inter-aural mismatch of 0, 2, or 6 mm between the left and right ears. Two FATs were tested: 1) “clinical FAT”, where the input acoustic frequency range was 200–8000 Hz in each ear, and 2) “matched FAT”, where the input frequency range was tonotopically matched to the simulated electrode position in each ear. Table 1 details the experimental parameters for the different insertion depths, with specific input and output frequency ranges for the clinical and matched FAT conditions. With the clinical FAT, the degree of tonotopic mismatch increased across the 27-, 25-, or 21-mm insertion depths. With the matched FAT, low frequency acoustic information was increasingly truncated across the 27-, 25-, or 21-mm insertion depths.

**Table 1:** Details of the 16-channel sinewave vocoders characteristics, including the simulated insertion depth in each ear, the experimental frequency allocation table (FAT) conditions, the input and output frequency ranges in each ear, the intra-aural mismatch within each ear at the most apical electrode, and the inter-aural mismatch across ears.

		Left ear			Right ear			
Insertion depth in mm (L, R)	FAT	Input range (Hz)	Output range (Hz)	Intra-aural mismatch at apical electrode in mm	Input range (Hz)	Output range (Hz)	Intra-aural mismatch at apical electrode in mm	Inter-aural mismatch in mm
27, 27	Clinical	200–8000	354–7771	2.7	200–8000	354–7771	2.7	0
27, 25	Clinical	200–8000	354–7771	2.7	200–8000	513–10290	4.7	2
27, 21	Clinical	200–8000	354–7771	2.7	200–8000	990–17990	8.7	6
27, 27	Matched	354–7771	354–7771	0	354–7771	354–7771	0	0
27, 25	Matched	354–7771	354–7771	0	513–10290	513–10290	0	2
27, 21	Matched	354–7771	354–7771	0	990–17990	990–17990	0	6

**Fig 1:**
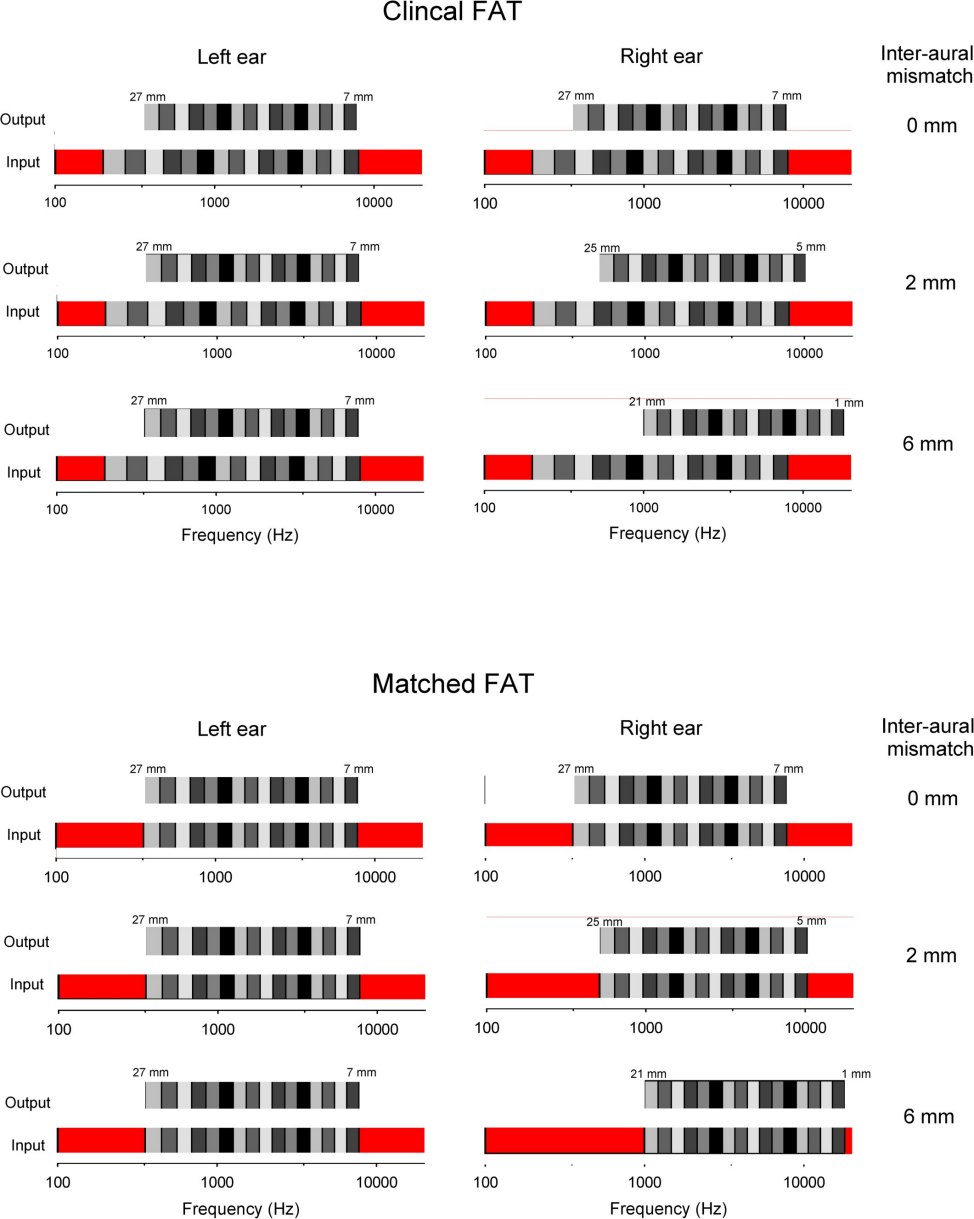
Illustration of the input and output frequency bands for the sinewave vocoders. Top: Input and output frequency ranges with the clinical FAT and the 27-, 25-, and 21-mm insertion depths. For the input frequency range, the red bars represent acoustic information loss due to the clinical FAT, and the grayscale bars represent the frequency analysis bands. The bottom axis shows the frequency, and the carrier. For the output frequency ranges, the grayscale bars represent the corresponding carrier bands; the sinewave carriers were centered within each carrier band. The cochlear location in mm from the base are shown above the carrier bands. Bottom: Same as the top, except for the matched FAT.

Fig 1 illustrates the input and output frequency bands for the left and right ears with the clinical and matched FATs, for the 27-, 25-, and 21-mm insertion depths. For the input frequency range with the clinical FAT, there is low frequency information loss <200 Hz and high frequency loss >8000 Hz (indicated by the red bars). The output frequency ranges shift towards the base as the insertion depth is reduced (i.e., increasing intra-aural mismatch in the right ear). For the input frequency range with the matched FAT, there is greater low-frequency information loss than with the clinical FAT: <354 Hz, <513 Hz, <990 Hz for the 27-, 25-, and 21-mm insertion depths, respectively. However, there is no intra-aural mismatch, even as the inter-aural mismatch increases.

### Test procedures

Testing was performed in a sound-attenuating booth. SRTs were measured using an adaptive procedure (1-up/1-down) that produced a 50% correct sentence recognition. The procedure was similar to a coordinate response matrix (CRM) test [10, 24]. Participants were instructed to listen to the target sentence (cued by the name “**John**”) and then click on one of the 10 response choices for each of the Number and Color categories; no other selections could be made from the remaining categories, which were greyed out. The target level was fixed at 65 dBA. The TMR was adjusted by varying the masker level from trial to trial according to the correctness of the responses for the target Number and Color keywords; the initial step size was 4 dB for the first two reversals in TMR, and the final step size was 2 dB. The SRT was calculated by averaging the last six reversals in TMR. The 12 listening conditions (2 FAT x 2 masker spatial configurations x 3 inter-aural mismatch) were randomized within a test block and 2–3 blocks were tested for each participant; SRTs for each condition were averaged across blocks. Participants were given no practice or previews prior to the testing sessions, and no feedback was provided during testing. All testing was completed in a single session with short breaks between test blocks.

### Data analysis

SRT data were analyzed using a linear mixed model (LMM), with FAT, spatial configuration type, and inter-aural mismatch, as fixed effects and participant as a random effect. The improvement in SRT data were also analyzed using a LMM, with benefit condition and inter-aural mismatch as fixed effects, and participant as a random effect. For the LMMs, significance was established for p < 0.05. Bonferroni correction was applied to post-hoc pairwise comparisons. LMMs were performed using SPSS (Version 20.0; Armonk, NY). All figures were generated using Sigmaplot software (Version 14).

## Results

**Fig 2:**
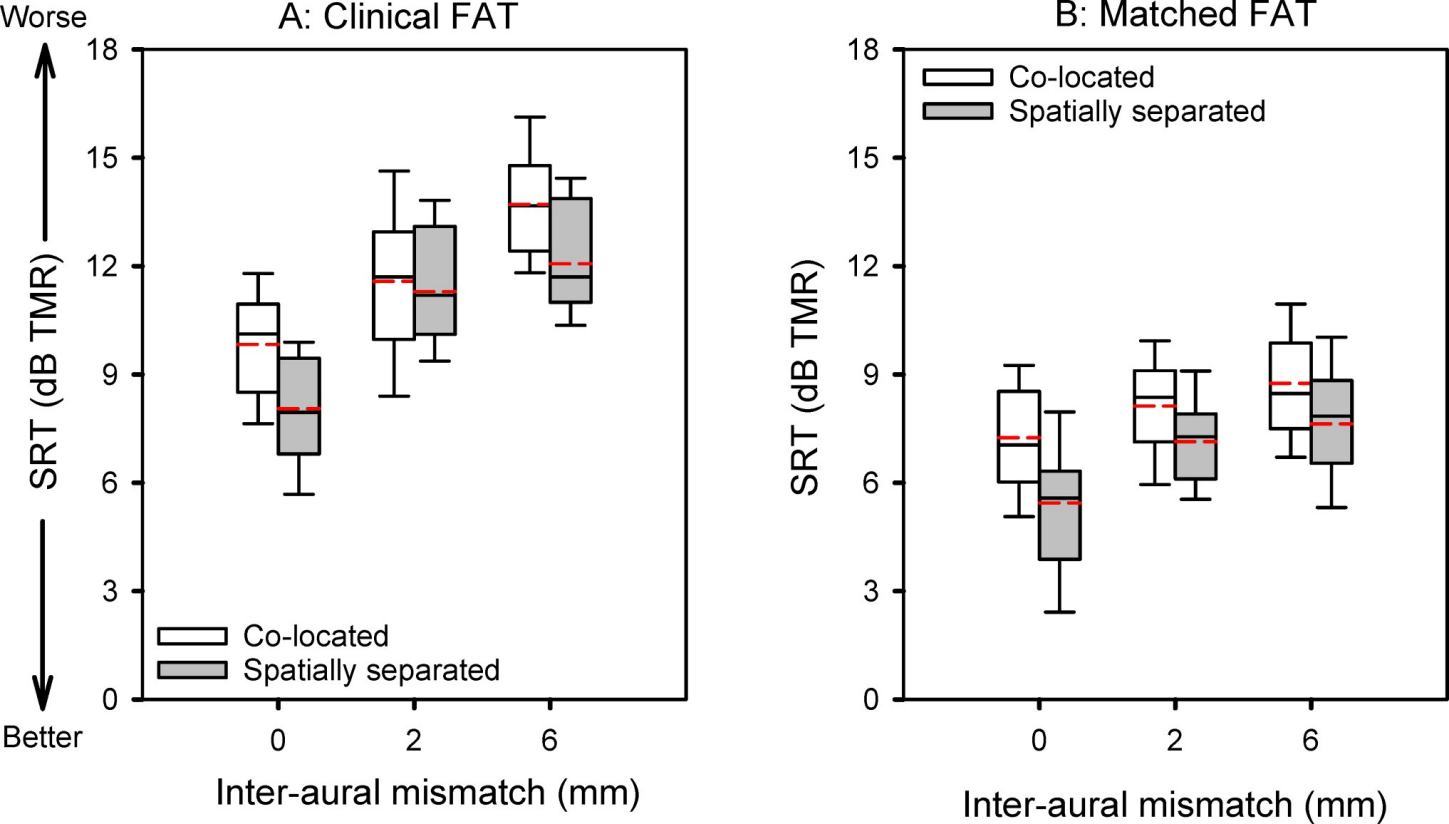
SRTs with the clinical FAT (Panel A) and matched FAT (Panel B), and with co-located or spatially separated target and masker speech, as a function of inter-aural mismatch. The boxes show the 25^th^ and 75^th^ percentiles, the error bars show the 10^th^ and 90^th^ percentiles, the symbols represent outliers, the solid horizontal line shows the median, and the dashed red line shows the mean.

Fig 2 shows SRTs with the clinical or matched FAT and with co-located or spatially separated target and masker speech, as a function of inter-aural mismatch. Mean SRTs are shown at the top of Table 2. In both the clinical and matched FAT conditions, SRTs progressively increased (worsened) with increasing inter-aural mismatch; SRTs were lower (better) with spatially separated target and masker speech. A linear mixed model (LMM) was performed on the SRT data, with FAT (clinical, matched), spatial configuration (co-located, spatially separated), and inter-aural mismatch (0, 2, or 6 mm) as fixed effects and participant as a random effect; complete results are shown at the bottom of Table 2. Significant effects were observed for FAT [F(1, 220) = 582.11, p < 0.001], spatial configuration [F(1, 220) = 68.71, p < 0.001], and inter-aural mismatch [F(2, 220) = 122.69, p < 0.001]. Significant interactions were observed between FAT and inter-aural mismatch [F(2, 220) = 15.64, p < 0.001], and between spatial configuration and inter-aural mismatch [F(2, 220) = 4.88, p = 0.008].

**Table 2:** SRTs for the FAT, spatial configuration, and inter-aural mismatch conditions. Top: SRTs (mean ± standard deviation) with the clinical or matched FAT, with co-located or spatially separated target and maskers, and with 0, 2, or 6 mm inter-aural mismatch. Bottom: Results from LMM analysis of the SRT data.

		Inter-aural mismatch		
FAT	Spatial configuration	0 mm	2 mm	6 mm
Clinical	Co-located	9.84±1.64	11.58±1.99	13.71±1.52
	Spatially separated	8.06±1.70	11.29±1.89	12.07±1.69
Matched	Co-located	7.25±1.41	8.13±1.42	8.76±1.51
	Spatially separated	5.44±1.83	7.14±1.33	7.64±1.59
**Fixed effects**	**dF, res**	***F***	***p***	**Post-hoc (*p* < 0.05)**
FAT	1, 220	582.11	*<0*.001*	Clinical >>> Matched
Spatial configuration	1, 220	68.71	*<0*.001*	Co-located >>> Spatial
Inter-aural mismatch	2, 220	122.69	*<0*.001*	6 >>> 2 >>> 0 mm
FAT x Spatial configuration	1, 220	0.04	0.834	
FAT x Inter-aural mismatch	2, 220	15.64	*<0*.001*	Clinical: 6 >>> 2 >>> 0 mm Matched: 6, 2 >>> 0 mm
Spatial configuration x Inter-aural mismatch	2, 220	4.88	*0*.008*	Clinical: 6 >>> 2 >>> 0 mm Matched: 6, 2 >>> 0 mm
FAT x Spatial configuration x Inter-aural mismatch	2, 220	1.33	0.267	

Asterisked and italicized values indicate significant effects. Significant differences from Bonferroni-adjusted pairwise comparisons are shown at right; “>>>” = *p* < 0.001; “>>” = *p* < 0.01; “>” = *p* < 0.05).

**Fig 3:**
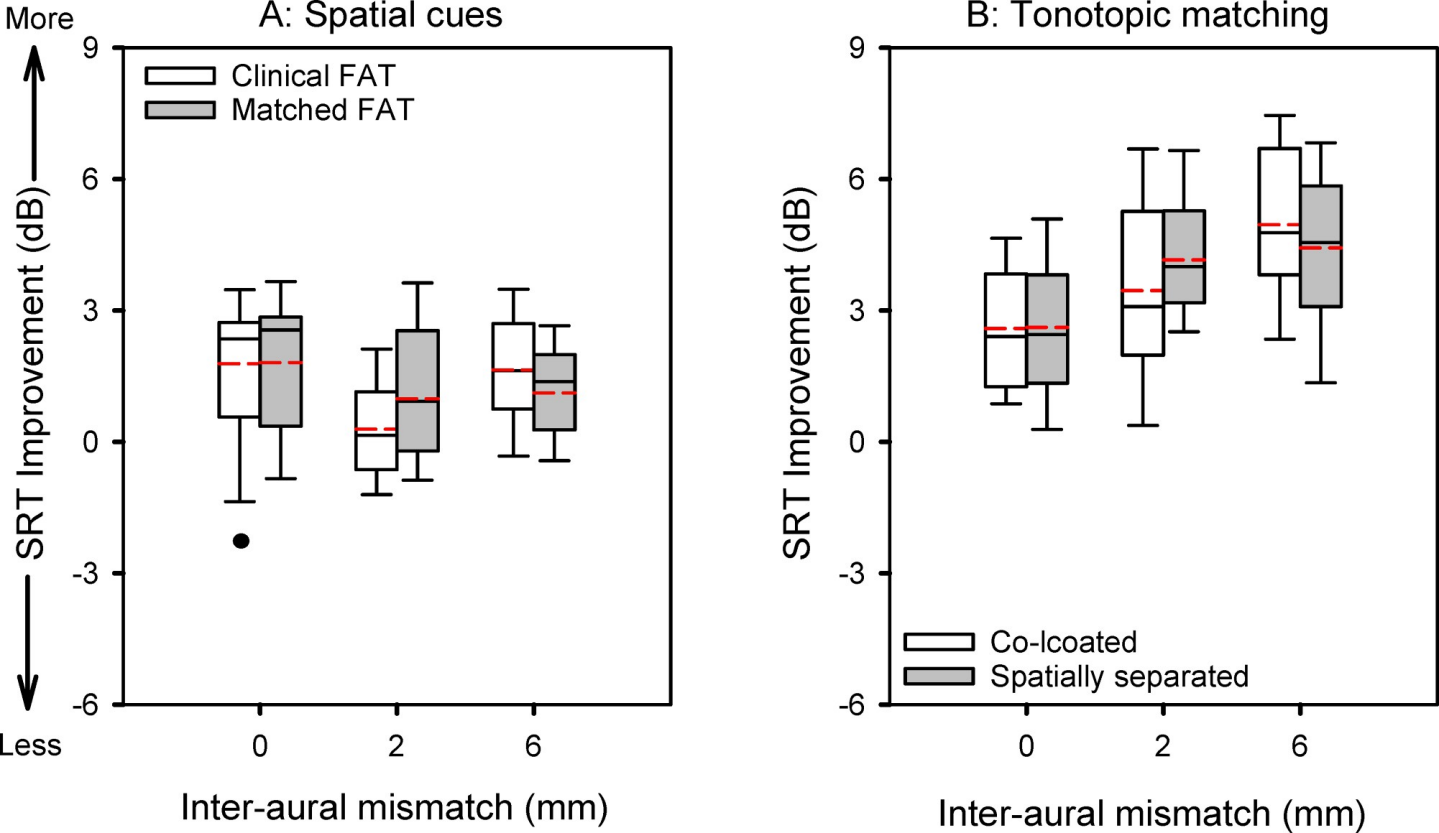
Boxplots of improvement in SRTs due to spatial cues (Panel A) or to tonotopic matching (Panel B), as a function of inter-aural mismatch. The boxes show the 25^th^ and 75^th^ percentiles, the error bars show the 10^th^ and 90^th^ percentiles, the symbols show outliers, the solid horizontal line shows the median, and the dashed red line shows the mean.

Fig 3 illustrates the improvement in SRTs due to spatial cues (co-located–spatially separated) or to tonotopic matching (clinical–matched FAT), as a function of inter-aural mismatch. Mean improvement in SRTs is shown at the top of Table 3. In general, the benefit of tonotopic matching was larger than that of spatial cues. The benefit of tonotopic matching was similar for co-located or spatially separated target and masker speech, and the benefit of spatial cues was similar with the clinical or matched FAT. LMM analysis was performed on SRT improvement data, with benefit condition (tonotopic matching for co-located target and masker speech, tonotopic matching for spatially separated target and masker speech, spatial cues with the clinical FAT, spatial cues with matched FAT) and inter-aural mismatch (0, 2, 6 mm) as fixed effects and participant as a random effect; complete results are provided at the bottom of Table 3. Significant effects were observed for benefit condition [F(3, 240) = 45.51, p < 0.001] and inter-aural mismatch [F(2, 220) = 7.06, p < 0.001]; there was a significant interaction [F(2, 240) = 6.05, p < 0.001]. Post-hoc Bonferroni adjusted pairwise comparisons showed that the benefit of tonotopic matching was significantly larger than that of spatial cues only for the 2 mm (p < 0.001) and 6 mm inter-aural mismatch conditions (p < 0.001). Interestingly, for spatial cues with the clinical FAT, the improvement in SRTs was significantly smaller for the 2 mm inter-aural mismatch than for the 0 mm (p = 0.011) and 6 mm mismatch conditions (p = 0.025).

**Table 3:** Improvement in SRTs due to spatial cues (co-located–spatially separated) or tonotopic matching (clinical–matched), as a function of inter-aural mismatch. Top: Improvement in SRTs (mean ± standard deviation) due to spatial cues with the clinical or matched FAT, or due to tonotopic matching with co-located or spatially separated target and masker speech. Bottom: Results from LMM analysis of the SRT improvement data.

**Benefit from spatial cues**				
		**Inter-aural mismatch**		
**FAT**		**0-mm**	**2-mm**	**6-mm**
Clinical		1.78±1.65	0.29±1.13	1.64±1.33
Matched		1.81±1.63	0.99±1.86	1.12±1.15
**Benefit from tonotopic matching**				
		**Inter-aural mismatch**		
**Spatial configuration**		**0-mm**	**2-mm**	**6-mm**
Co-located		2.59±1.65	3.46±2.08	4.96±1.87
Spatially separated		2.61±1.73	4.15±1.57	4.43±1.87
**Fixed effects**	**dF, res**	***F***	***p***	**Post-hoc (*p* < 0.05)**
Condition	3, 240	45.51	*<0*.001*	Tonotopic matching (co-located, spatially separated) >>> Spatial cues (clinical, matched)
Insertion	2, 240	7.06	*<0*.001*	6 >> 2, 0 mm
Condition x Insertion	6, 240	6.05	*<0*.001*	2, 6 mm: Tonotopic matching (co-located, spatially separated) >>> Spatial cues (clinical, matched)
				Spatial cues (clinical): 0, 6 > 2 mm
				Tonotopic matching (co-located): 6 > 0, 2 mm
				Tonotopic matching (spatial): 2, 6 >> 0 mm

Asterisked and italicized values indicate significant effects. Significant differences from Bonferroni-adjusted pairwise comparisons are shown at right; “>>>” = *p* < 0.001; “>>” = *p* < 0.01; “>” = *p* < 0.05).

## Discussion

The present bilateral CI simulation data show that binaural SRTs worsened with increasing inter-aural mismatch for both co-located and spatially separated target and maskers (Fig 2, Panel A). Reducing intra- and inter-aural mismatch by adjusting the FAT significantly improved segregation of competing speech, with or without spatial cues. The present data suggest a beneficial tradeoff between tonotopic matching and low frequency information loss for bilateral CI users, especially when there is a substantial difference in insertion depth across ears.

### SRTs and masking release with the clinical FAT

With 0-mm inter-aural mismatch, the mean SRT for the co-located maskers was 9.8 dB, similar to Xu et al. [10]. Mean SRTs significantly worsened by 1.7 dB when the inter-aural mismatch was increased from 0 to 2 mm, also consistent with Xu et al. [10]. Mean SRTs significantly worsened by 2.1 dB when the inter-aural mismatch was further increased from 2 to 6 mm. This is similar to the results from Yoon et al. [25], who investigated the effects of binaural spectral mismatch on binaural benefit (the binaural advantage over the better ear alone) for speech understanding in steady noise, using acoustic simulations of bilateral CIs in NH listeners. As in the present study, binaural spectral mismatch was systematically manipulated by varying the relative insertion depth across ears. They reported significant binaural benefits when the inter-aural mismatch was 1 mm or less. When the inter-aural mismatch was 2 mm or more, including the ear with the shallower insertion typically resulted in a negative binaural benefit. Yoon et al. [25] suggested that adding the CI with the shallower insertion may interfere with the benefits provided by the CI with a deeper insertion.

**Fig 4:**
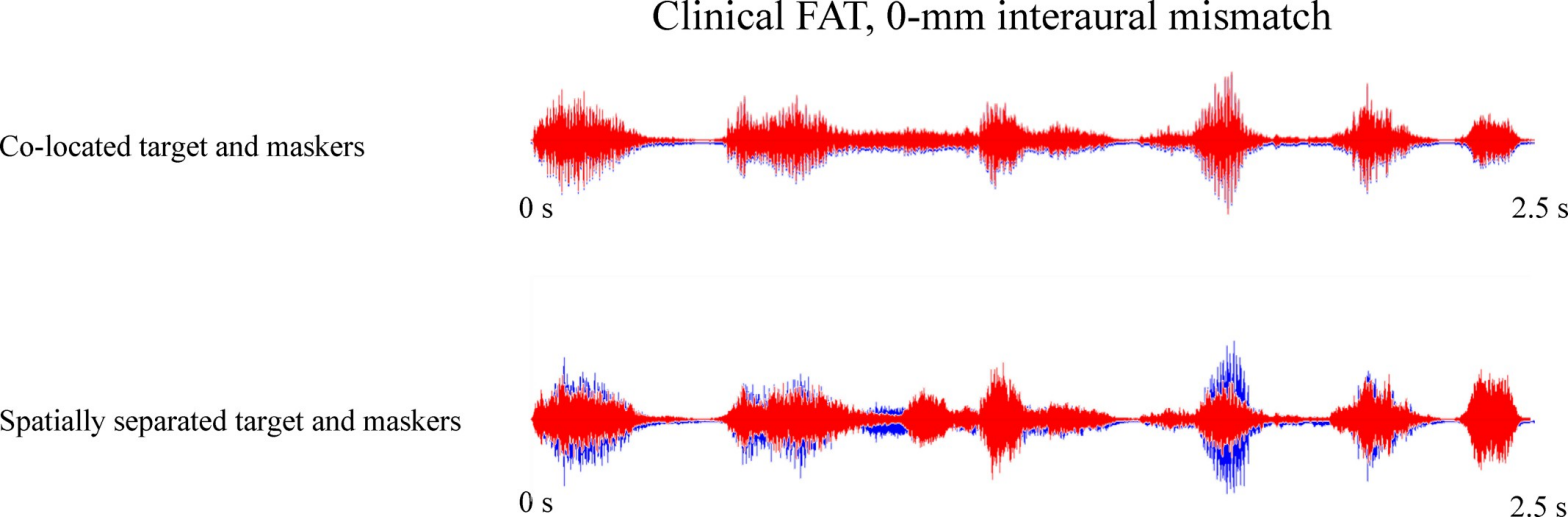
Combined target with co-located or spatially separated maskers. The TMR was 0 dB. Waveforms are shown after HRTF and the bilateral CI simulation was applied. The blue and red traces show the waveforms presented to left and right ears, respectively. The left and right waveforms are slightly offset for visual purposes.

With the spatially separated maskers, SRTs worsened by 3.2 dB with 2-mm inter-aural mismatch, much less than the drop of 7.5 dB for the dichotic masker presentation in Xu et al. [10]. This is consistent with the observation that dichotic presentation may overestimate spatial benefits for the segregation of competing speech [13–17, 19]. Accordingly, the 1.8 dB of spatial masking release was also much less than the 7.6 dB observed in Xu et al. (2020). SRTs worsened by 1.2 dB (not significant) when the inter-aural mismatch was increased from 2 to 6 mm, less than the deficit observed when the inter-aural mismatch was increased from 0 to 2 mm (3.2 dB). This suggests that adding the CI with very shallow insertion depth was less detrimental for segregation of spatially separated speech than for segregation of co-located speech. Such differential effects may be driven by the degree of similarity of the temporal envelope across ears. Fig 4 shows waveforms for combined target and maskers at 0 dB TMR with the clinical FAT, after processing by the HRTF and the bilateral CI simulation. For co-located speech, the temporal envelope was exactly the same in both ears (i.e., co-modulated). For spatially separated speech, the temporal envelope differed across ears. The modulation detection threshold of a target carrier has been shown to be adversely affected by the presence of other modulated carriers, especially when the target and interfering carriers were co-modulated [26]. Modulation detection thresholds have been significantly correlated with CI users’ speech recognition performance [27]. Overall, the results suggest that speech information from the poorer ear might provide less interference to recognition of target speech in the better ear when spatial cues are available, thus increasing spatial masking release as the inter-aural mismatch was increased.

### SRTs and masking release with the matched FAT

For the clinical FAT, the intra-aural tonotopic mismatch increased as the insertion depth was reduced in the right ear. As shown in Fig 1 and Table 1, the low-frequency information loss was <200 Hz. For the matched FAT, there was no intra-aural mismatch. However, the low-frequency information loss was greater than with the clinical FAT, and increased as the insertion depth was reduced (<354 Hz, <513 Hz, and <990 Hz for the 27-, 25-, and 21-mm insertion depths, respectively). As the low-frequency input frequency is increased, the information loss increases, which negatively affects speech recognition performance [28, 29].

For the co-located maskers, mean SRTs with the matched FAT were 7.3, 8.1, and 8.7 dB TMR for the 0-mm, 2-mm, and 6-mm inter-aural mismatch conditions, respectively, much lower than SRTs with the clinical FAT. Tonotopic matching within each ear improved mean SRTs by 2.6, 3.5, and 5.0 dB as the inter-aural mismatch was increased from 0 to 2 to 6 mm, respectively. These results suggest that, when tonotopically matched within each ear, spectral cues from the ear with the shallower insertion depth might not interfere with the recognition of target speech in the ear with the deeper insertion, despite the greater information loss.

With the spatially separated maskers, mean SRTs with the matched FAT were 5.4, 7.1, and 7.6 dB TMR for the 0-mm, 2-mm, and 6-mm inter-aural mismatch conditions, respectively. As with the co-located maskers, SRTs with spatially separated maskers were much lower with the matched FAT than with the clinical FAT. Tonotopic matching within each ear improved mean SRTs by 2.6, 4.2, and 4.4 dB as the inter-aural mismatch was increased from 0 to 2 to 6 mm, respectively. There was no significant difference in the benefit of tonotopic matching within each ear when maskers were co-located or spatially separated.

While the benefit of spatial cues was similar with the clinical and matched FATs, the benefit of tonotopic matching was much larger than the benefit of spatial cues (top of Table 2). This likely is due to limited benefit of spatial cues when spectral resolution is reduced, as observed with bilateral CI users and bilateral CI simulations [10, 19, 20].

### Clinical relevance and study limitations

The bilateral CI simulation data collected in this study are consistent with Svirsky et al. [8], who showed a significant benefit of tonotopic matching in bilateral CI users with large inter-aural mismatch. With a moderate inter-aural mismatch (2 mm), tonotopic matching was more beneficial for spatially separated than for co-located maskers. This suggests that a mild or moderate inter-aural mismatch might negatively affect the utilization of available spatial cues for the segregation of competing speech. The present bilateral CI simulation data suggest that inter-aural mismatch may underlie the poor (or even negative) spatial masking release observed in real bilateral CI listeners [19, 20].

There are some limitations and caveats to the present study that should be considered. First, the NH participants presumably had uniform nerve survival within the carrier frequency ranges tested. This is not likely to be the case in real bilateral CI users; even when there is no difference in electrode position across ears, there may be uneven nerve survival patterns within each ear, resulting in inter-aural mismatch in terms of the electrode-neural interface. Second, performance was acutely measured in the present NH listeners with no training, feedback, or practicing experience with the various CI simulations. In the case of real CI users, long-term experience may mitigate some of the effects of intra- and inter-aural frequency mismatch due to neuroplastic adaptation over time. However, it still may be advantageous to reduce inter-aural mismatch in bilateral CI users, especially during the initial usage period with the device [8]. Thus, if the degree of inter-aural mismatch can be ascertained in bilateral CI users, adjustment to the FAT in either ear can significantly enhance the successful segregation of competing speech.

### Summary and conclusion

The present study explored the trade-off between reduced intra-aural frequency mismatch and the loss of low-frequency information for the segregation of competing speech in normal-hearing (NH) participants listening to acoustic simulations of bilateral CIs where differences in insertion depth were varied. Major findings include:

- The matched FAT provided better SRTs than the clinical FAT, regardless of the insertion depth or spatial configuration. This suggests a strong benefit for tonotopic matching within and across ears.
- Compared to the clinical FAT, the matched FAT provided the largest improvement in SRTs when there was a large difference in insertion depth (6 mm). Moreover, spatial masking release significantly depended on the difference in insertion depth across ears for the clinical FAT, but not for the matched FAT.
- Minimizing intra-and inter-aural mismatch by adjusting the FAT may benefit overall SRTs, especially when there is a large difference in insertion depth across ears. Minimizing intra-aural mismatch may also improve masking releases when there is a moderate difference in insertion depth across ears.
- These tonotopic matching benefits were observed despite substantial low-frequency information loss in the CI ear with a very shallow insertion depth. Estimates of electrode positions in each ear may guide the implementation of optimal FATs that can improve the ability of bilateral CI users to segregate competing speech with or without spatial cues.

## Supporting information

S1 Data (XLSX)
